# Supplementary material for: The interactome of intact mitochondria by cross-linking mass spectrometry provides evidence for coexisting respiratory supercomplexes
Source: Mol Cell Proteomics. 2017 Dec 8;17(2):216–32. doi: 10.1074/mcp.RA117.000470 (PMC5795388; doi:10.1074/mcp.RA117.000470)
Supplement: Supplemental Data [file supp_RA117.000470_133922_0_supp_23978_fzffwf.zip › spectra_annotation/mito_spectra_annotation/227-1-10-1-12-1.pdf]

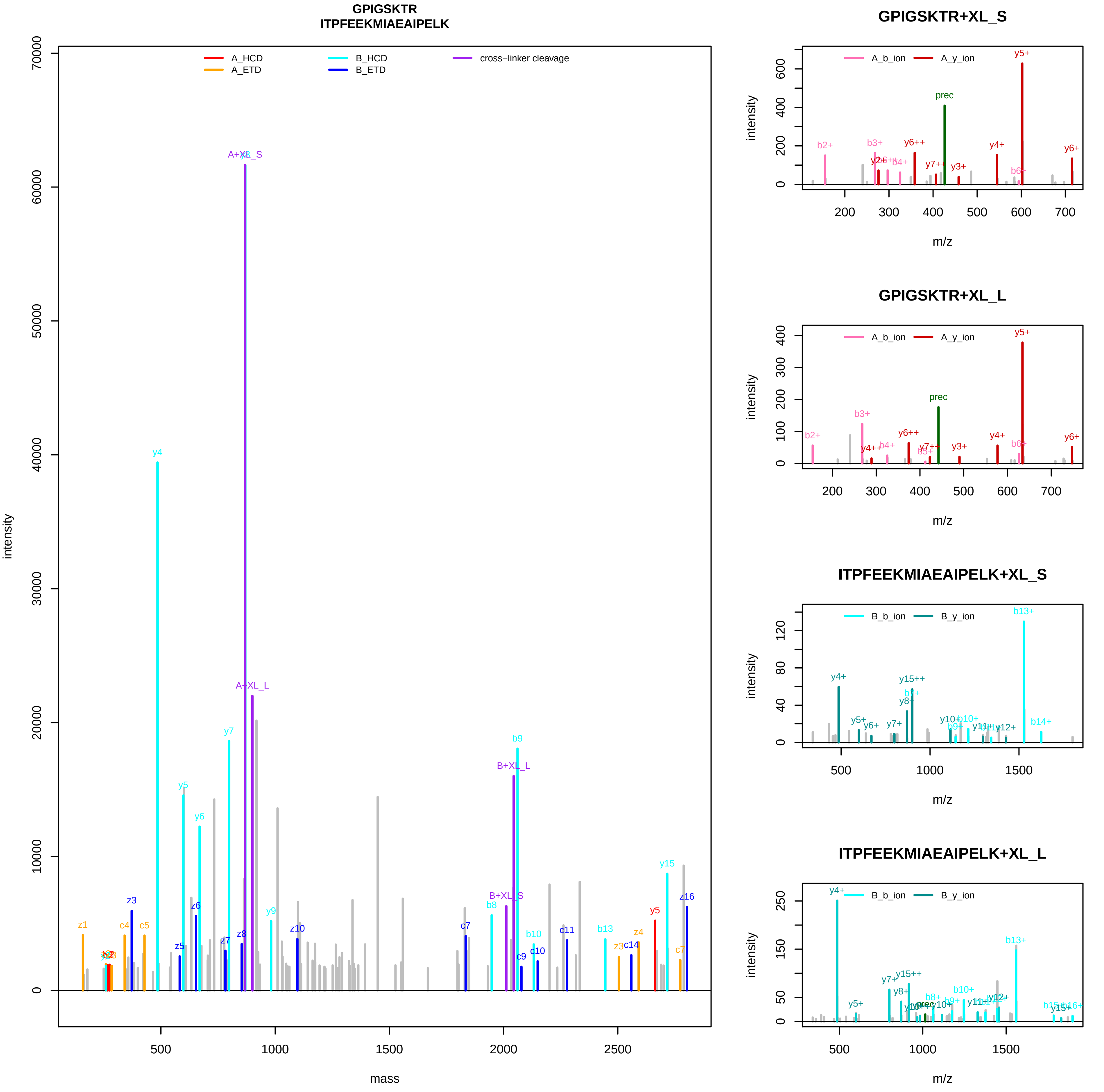
GPIGSKTR
ITPFEEKMIAEAIPELK
A_HCD
A_ETD
B_HCD
B_ETD
cross−linker cleavage
A+XL_S
A+XL_L
B+XL_S
B+XL_L
y4
y5
y6
y7
y9
y15
b8
b9
b10
b13
z1
z3
z5
z6
z7
z8
z10
z16
c4
c5
c7
c9
c10
c11
c14
z4
y5
intensity
mass
GPIGSKTR+XL_S
GPIGSKTR+XL_L
ITPFEEKMIAEAIPELK+XL_S
ITPFEEKMIAEAIPELK+XL_L
A_b_ion
A_y_ion
B_b_ion
B_y_ion
prec
m/z
